# MicroRNA-149* suppresses hepatic inflammatory response through antagonizing STAT3 signaling pathway

**DOI:** 10.18632/oncotarget.18541

**Published:** 2017-06-16

**Authors:** Qiqi Zhang, Jia Su, Ziwei Wang, Hui Qi, Zeyong Ge, Zhijun Li, Wei-Dong Chen, Yan-Dong Wang

**Affiliations:** ^1^ State Key Laboratory of Chemical Resource Engineering, College of Life Science and Technology, Beijing University of Chemical Technology, Beijing, P. R. China; ^2^ Key Laboratory of Receptors-Mediated Gene Regulation and Drug Discovery, School of Medicine, Henan University, Kaifeng, P. R. China; ^3^ Key Laboratory of Molecular Pathology, School of Basic Medical Science, Inner Mongolia Medical University, Hohhot, P. R. China

**Keywords:** miR-149*, liver inflammation, STAT3, miRNA, CRISPR/CAS9

## Abstract

Chronic inflammation is increasingly recognized as an important component of tumorigenesis and metabolic diseases. The roles of microRNA149* (miRNA149*) in inflammation remain poorly understood. Here, we demonstrate that miR-149* is a suppressor of STAT3-mediated inflammation. MiR-149*^−/−^ mice were generated with CRISPR/CAS9 technique. In a lipopolysaccharide (LPS)-induced inflammation model, miR-149*^−/−^ mice show more severe liver injury and inflammation, compared with wild-type (WT) mice. MiR-149*^−/−^ mice also displayed elevated messenger RNA (mRNA) levels of interleukin (IL)-6, inducible nitric oxide synthase (iNOS), complement C3 (C3) and IL-4 in response to LPS. Then miR-149* agomir administration is largely able to alleviate the LPS-induced some inflammatory gene expression in WT mouse liver. In vitro, miR-149* mimics inhibited expression of STAT3-meidated inflammatory mediators induced by LPS and suppresses the phosphorylation of STAT3 and its transcription activity in HepG2 cells. These findings identify miR-149* as a negative mediator of inflammation that may serve as an attractive therapeutic tool for immune and inflammatory liver diseases.

## INTRODUCTION

Inflammatory responses are very important in regulating liver pathological conditions [1, 2]. Chronic inflammation has been shown to play important roles in tumorigenesis and metabolic diseases [3]. For example, chronic hepatitis often leads to hepatocellular carcinoma, a prototype of inflammation-associated cancer [4]. Thus, precisely controlling inflammation is necessary for inhibiting the progression of diseases such as atherosclerosis and cancer [5, 6].

MicroRNAs (miRNAs) represent distinct small noncoding RNAs of about 22 nt long that regulate gene expression posttranscriptionally [7]. miRNA can silence gene expression through binding to 3′UTR as well as 5’UTR or even coding regions of target mRNAs [8, 9]. Patients and animal models of autoimmune diseases show dysregulated miRNA expression, and functional studies have pinpointed the essential roles of miRNAs in the onset and development of autoimmune diseases such as multiple sclerosis (MS) and rheumatoid arthritis (RA) [10–12]. The importance of miRNAs in the liver has been studied by different groups. For example miR-122 regulates hepatic cholesterol and lipid metabolism, thereby having a central role in maintaining liver homeostasis [13]. Bala et al. reported that miR-155 promotes alcohol-induced steatohepatitis and fibrosis *in vivo*. Increasing studies have suggested that miRNAs are critical regulators of development and function in liver inflammation-related diseases.

Pre-miRNAs are cleaved by RNase III type enzyme, Dicer, to generate an approximately 22-nt miRNA duplex: one strand (miRNA*) of the duplex is often degraded shortly, whereas the other strand serves as a mature miRNA [14]. However, multiple reports suggest that not all miRNA*s are short-lived. Some of them efficiently regulate gene expression as its complementary mature miRNAs do [15]. miRNA-149 (miR-149) became a focus in some studies because of its essential functions in suppressing cancer cell proliferation and migration and inflammatory response, while there are few reports about the functions of miRNA-149* in physiological condition except for the reports of Ding et al. and Jin et al. [14, 16]. The function of miR-149* in inflammatory response are still not known.

In this article, using miR-149* knockout mice generated by CRISPR/CAS9 technique and cell culture system, we identify miR-149* as a negative regulator of inflammatory response. We demonstrate that the deficiency of miR-149* *in vivo* are more sensitive to LPS-induced mouse liver inflammation and injury. Moreover, we show that the regulation of miR-149* on inflammatory response is associated with deactivation of STAT3 cell signaling. Thus, miR-149* may be a potential therapeutic target for treatment of inflammation.

## RESULTS

### Generation of miR-149*^−/−^ mice by CRISPR/Cas9 system

**Figure 1:**
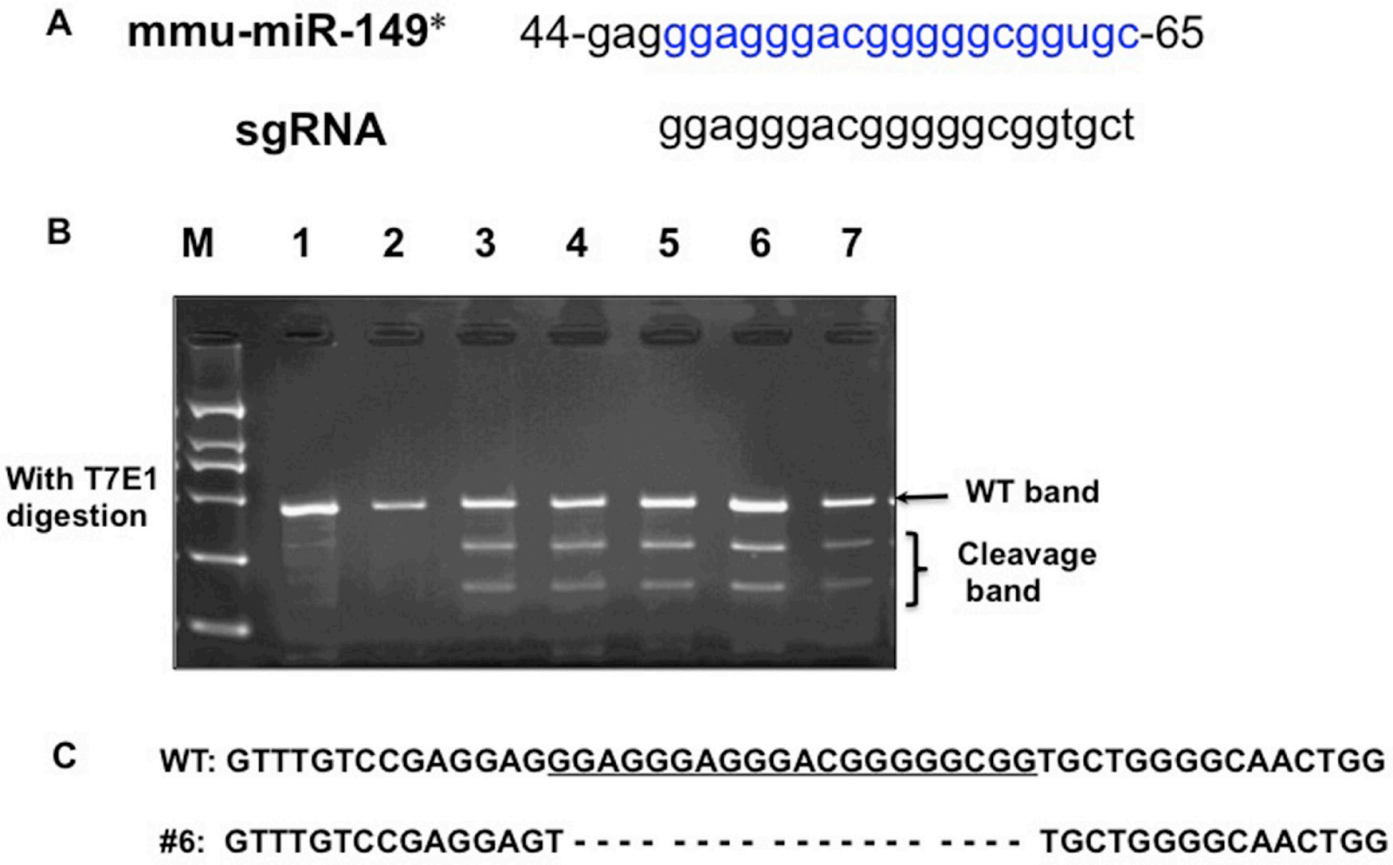
Generation of miR-149* mutant mice by using the CRISPR/Cas9 system (**A**) Schematic of the mouse miR-149* sequence (mmu-miR-149*) and the binding sites of the sgRNA. (**B**) Detection of mutations in F0 mice by T7E1 digestion using PCR products amplified from ear genomic DNA. Cleavage bands indicate the presence of mutations in the miR-149* gene in F0 mice. (**C**) The DNA sequence of the mutant miR-149* gene in founder #6 of F1 mice. Four TA clones of the PCR products amplified from each founder were analyzed by DNA sequencing. sgRNA sequences are underlined. The short dash lines indicate deletion of nucleotides.

To generate a miR-149*-null allele, one single guide RNAs (sgRNA) targeting miR-149* of the mouse gene (Figure 1A) was co-injected with Cas9 mRNA into the cytoplasm of fertilized eggs with well recognized pronuclei in M2 medium (Sigma). 15–25 blastocysts were then transferred into uterus of pseudopregnant females. Genomic DNA from the newborns was extracted from mouse ears for PCR amplification using specific primers (F, CTGTTCTGATGTTGAGCACCTATGG; R, GGCAGGTTCTGGATAAATGGGAC). The PCR products were used for the T7 Endonuclease I (T7EI) assay. After T7EI digestion, 5 out of 12 pups were identified as F0 founders, bearing mutations in the miR-149* (Figure 1B). For sequencing, the PCR products of genome modification which was detected by T7EI assay were cloned using TA cloning Kit, and mutations were identified by Sanger sequencing. DNA sequencing of the 5 founders confirmed that these were heterozygotes with deletion mutations in one allele. Among these, the 19 base pairs of miR-149* DNA was deleted in the mutation in founder #6 (Figure 1C). DNA sequencing confirmed that the same miR-149* mutation was present in the F1 mice, thereby suggesting that this mutated miR-149* allele is heritable. We bred founder #6 with WT mice for at least two generations, and miR-149*^+**/**−^ mice were then bred to homozygotes.

### miR-149*^−/−^ mouse hepatic tissue display increased expression of proinflammatory genes

**Figure 2:**
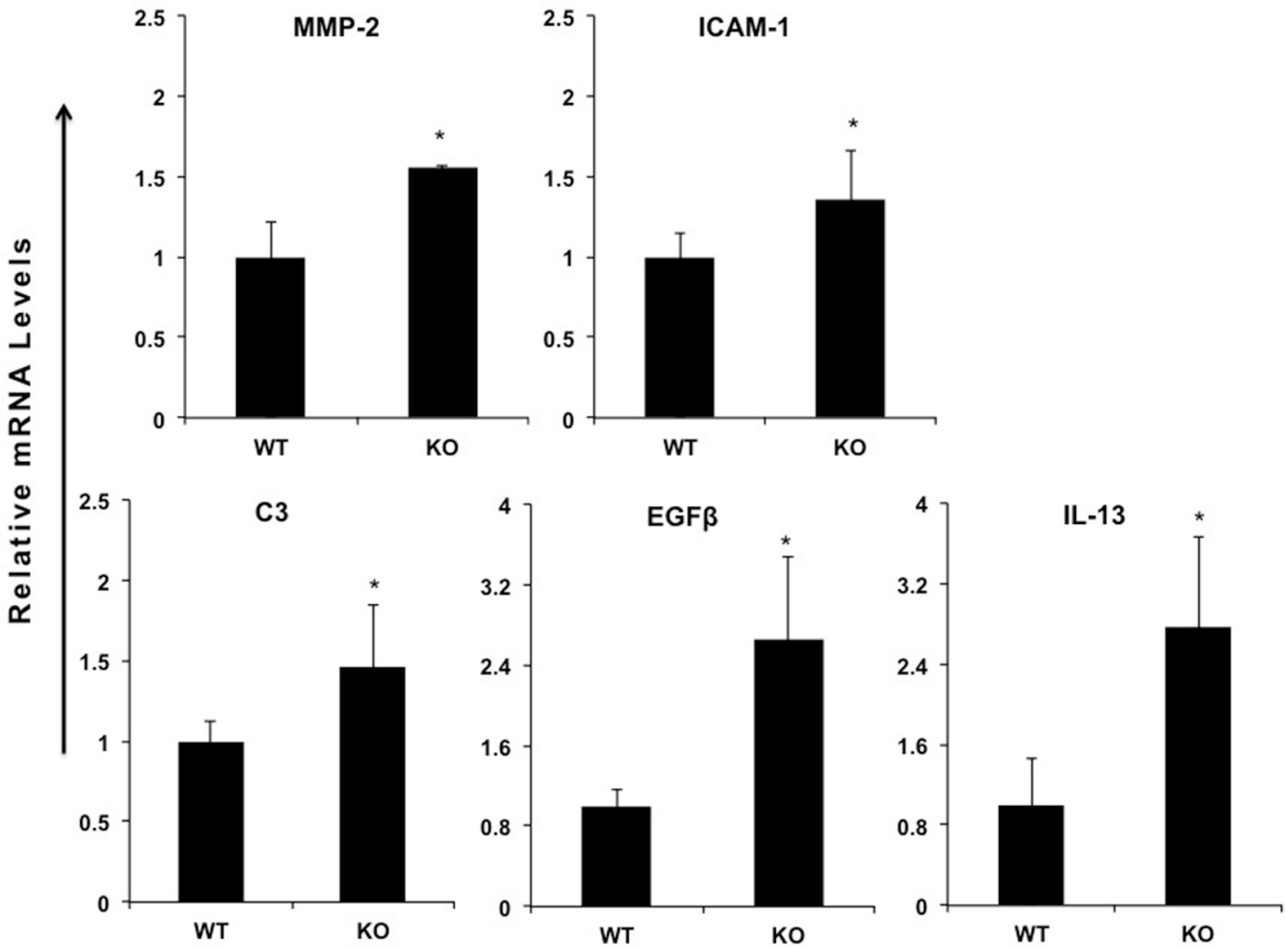
miR-149*^−/−^ mouse hepatic tissue display increased expression of proinflammatory genes Quantitative real-time PCR analysis of the expression of proinflammatory genes in livers from 8-week-old wild-type (WT) or miR-149*^−/−^ (KO) mice (*n* = 5). *P < 0.05 versus the WT group.

In order to investigate the function of miR-149* in inflammatory response, we determined the expression of proinflammatory genes in WT and miR-149*^−/−^ mouse liver. We found that, compared with WT controls, livers from miR149*^−**/**−^ mice had increased mRNA levels of some genes associated with inflammation (Figure 2). These increased genes include matrix metalloproteinase-2 (MMP2), intercellular cell adhesion molecule-1 (ICAM-1), complement component 3 (C3), epidermal growth factor β (EGFβ) and IL-13 (Figure 2). Moreover, we found these genes are the target genes of STAT3, which indicates that miR-149* may be a regulator of STAT3 signaling pathway.

### The deficiency of miR-149* in mouse liver is more sensitive to LPS-induced inflammation and injury

**Figure 3:**
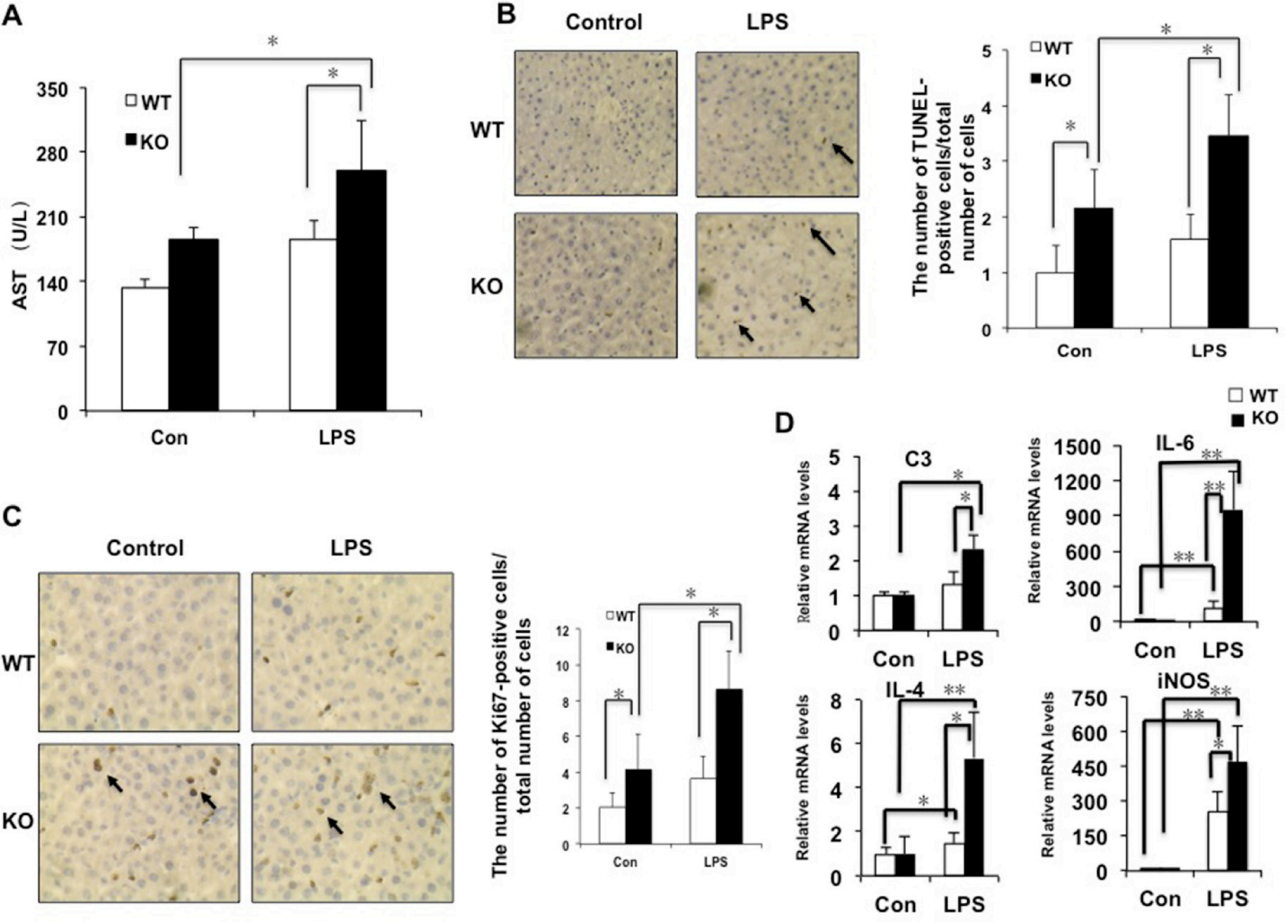
The deficiency of miR-149* in mouse liver is more sensitive to LPS-induced liver injury 8-week-old WT and miR-149*^−/−^ (KO) mice were treated with LPS (10mg/Kg body weight) for 16 hours. Then the mouse livers and blood were collected for further analysis. (**A**) AST levels of wild-type (WT) and miR-149*^−/−^ (KO) mice after LPS treatment (*n* = 5–6). Con, control; LPS, LPS-treated groups. *P* < 0.05. (**B**) Representative TUNEL staining of sections from WT and miR-149*^−/−^ livers (magnification X200) and statistical analysis of the number of TUNEL-positive cells per total number of cells. The number of cells in at least 20 microscopic fields was counted. *P* < 0.05 (*n* = 5–6). Black arrows, TUNEL-positive cells. (**C**) Representative Ki67 staining of sections from WT and miR-149*^−/−^ livers (KO) (magnification ×200) and statistical analysis of the number of Ki67-positive cells per total number of cells. The number of cells in at least 20 microscopic fields was counted. *P < 0.05. Black arrows, Ki67-positive cells. (D) Quantitative real-time PCR (qRT-PCR) analysis of the expression of proinflammatory genes in livers from WT or miR-149*^−/−^ (KO) mice (*n* = 5–6). *P < 0.05 and **P < 0.05.

In order to test whether miR-149* is an inhibitor of inflammation, we investigated the levels of aspartate aminotransferase (AST), a marker of liver damage, after LPS treatment. LPS treatment significantly increased AST levels in miR-149*^−/−^ mice but not in WT mice (Figure 3A). After LPS treatment, AST levels in miR-149*^−/−^ mice were higher than that in WT mice. TUNEL assays were used for testing LPS-induced liver injury. MiR-149*^−/−^ control mouse livers displayed higher TUNEL-positive cell staining than that of WT control groups. Moreover, LPS administration induced considerable TUNEL-positive staining in the livers of miR-149*^−/−^ mice, compared with that in wild-type mice (Figure 3B). The results reveal that miR-149*^−/−^ mice are more sensitive to LPS-induced liver injury.

LPS can cause the liver proliferative response [17]. We next examined hepatocyte proliferative response post LPS-induced injury by Ki67 staining. Considerable Ki67-positive cells were detected in miR-149*^−/−^ mouse liver compared with WT groups, even without LPS treatment (Figure 3C). LPS administration increased the numbers of Ki67-positive cells with 0.8 folds and 1.1 folds in WT and miR-149*^−/−^ mouse livers, respectively. It suggested that LPS increased Ki67-positive cell number more significantly in miR-149*^−/−^ mouse liver.

We compared proinflammatory gene expression in livers between miR-149*^−/−^ and WT mice after LPS administration. The mRNA levels of C3, IL-6, IL-4 and iNOS induced by LPS was greater in miR-149*^−/−^ mice than that in WT mice (Figure 3D). The results indicate that certain inflammatory genes in the deficiency of miR-149* in mouse liver are more sensitive to LPS induction.

### miR-149* regulates liver inflammatory response *in vivo* and *in vitro*

**Figure 4:**
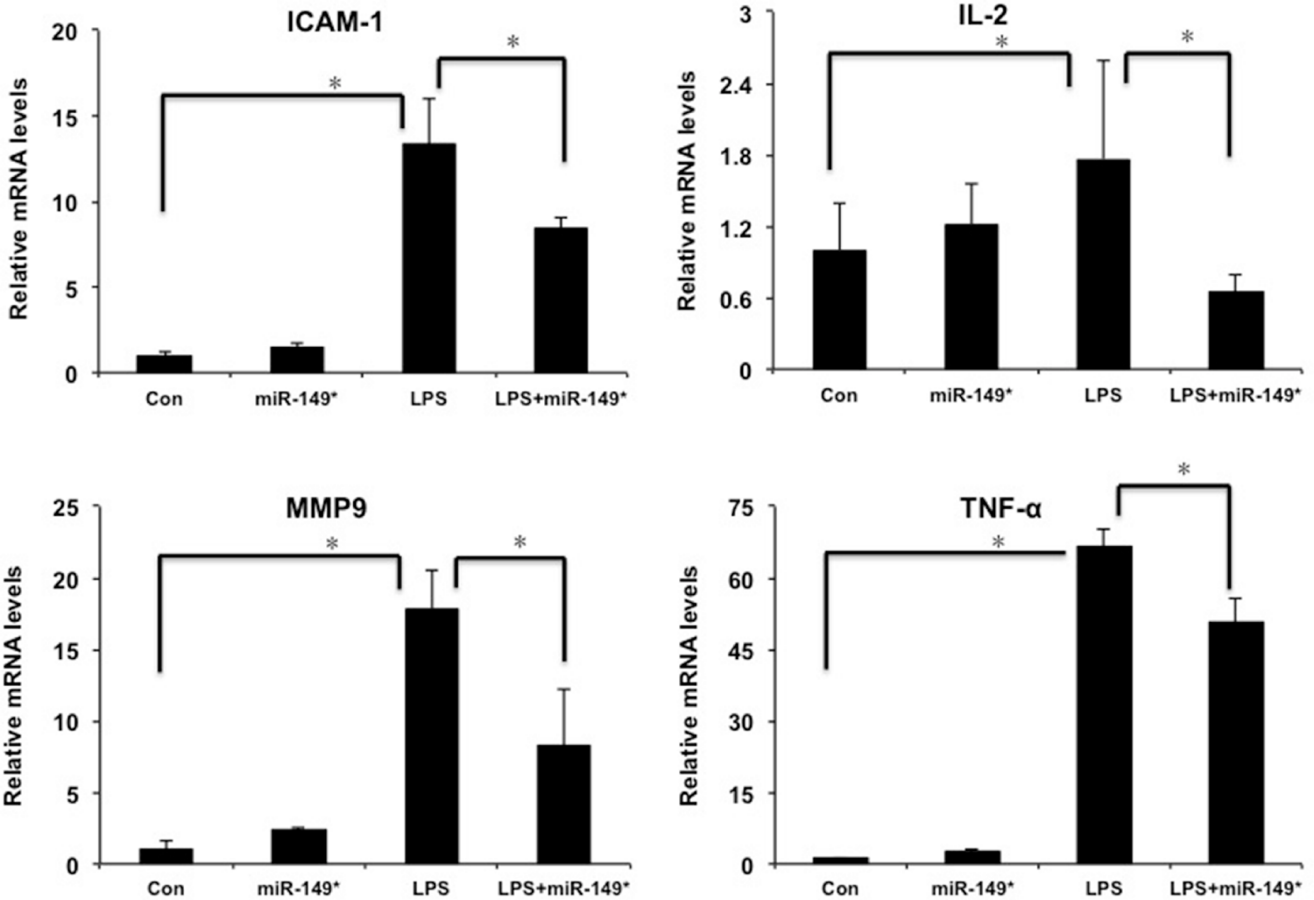
miR-149* suppressed inflammatory gene expression induced by LPS in mouse liver QRT-PCR analysis of the expression of proinflammatory genes in mouse liver. WT mice were injected in the tail vein with miR-149* agomir or negative control agomir. Two day later, mice were treated with LPS (20 mg/Kg body weight) for 6 hours. Then the mouse livers were collected for qRT-PCR analysis. (*n* = 4–5) *p < 0.05. Con, negative control agomir-treated group; miR-149*, miR-149* agomir-treated group; LPS, negative control agomir+LPS-treated group; LPS+miR-149*, LPS+miR-149* agomir-treated group.

In order to investigate the function of miR-149* in liver inflammatory response *in vivo*, we investigated the role of miR-149* agomir in liver inflammation induced by LPS. LPS obviously increased the mRNA levels of ICAM-1, IL-2, MMP9 and TNF-α (Figure 4). The agomir of miR-149* reduced LPS-induced the expression of ICAM-1, IL-2, MMP9 and TNF-α (Figure 4).

**Figure 5:**
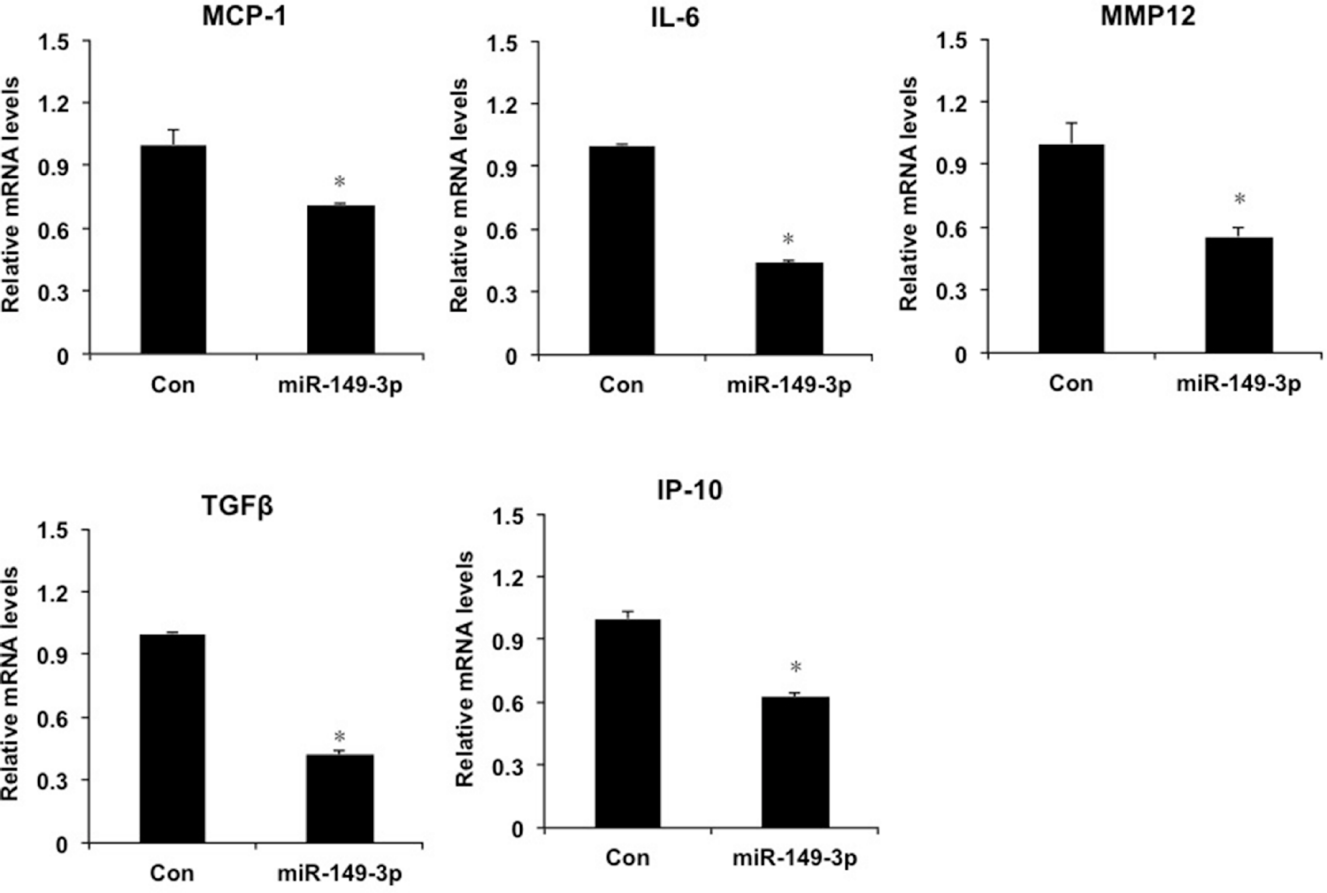
miR-149* regulates liver cell inflammatory response in HepG2 cells QRT-PCR analysis of the expression of proinflammatory genes in HepG2 cells. Cells were transfected with miR-149* mimics (25 nM) or negative control mimics. Twenty-four hours, cells were collected and RNA was extracted for qRT-PCR analysis. (*n* = 3) *P* < 0.05 versus the control groups. Con, negative control mimics; miR-149-3p, miR-149* mimics.

Furthermore, we investigated the role of overexpression of miR-149* mimics in HepG2 cells. Compared with the mRNA levels of proinflammatory genes in control group, miR-149* mimics resulted in the decrease of mRNA levels of MCP-1, IL-6, MMP12, TGFβ and IP-10 (Figure 5), which suggests that miR-149* suppresses liver cell inflammatory response *in vitro*.

**Figure 6:**
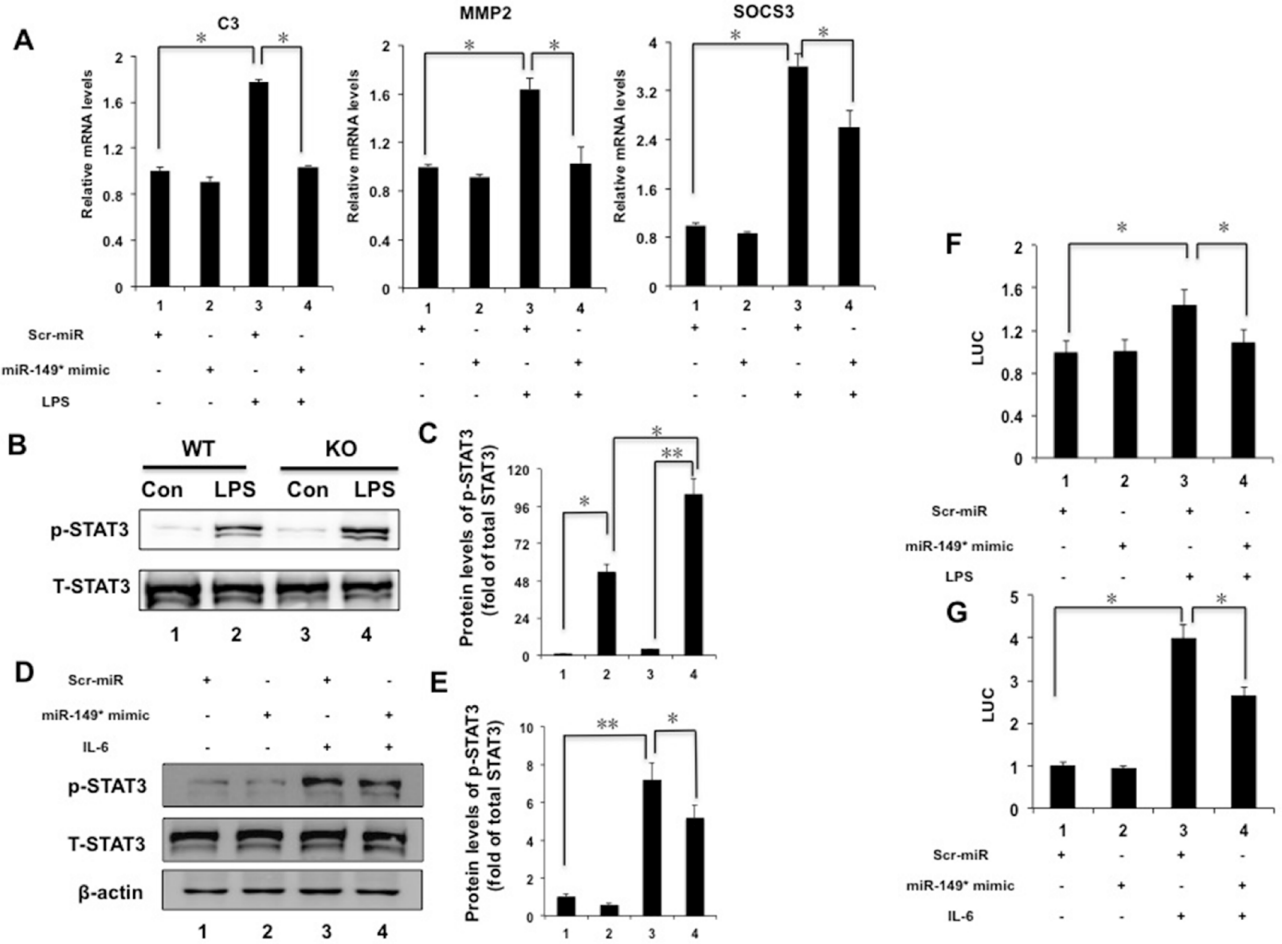
miR-149* suppressed STAT3 cell signaling pathway (**A**) miR-149* suppressed STAT3-meidated target genes induced by LPS. HepG2 cells were transfected with miR-149* mimics or control mimics (Scr-miR). After 24 hours, cells were treated with LPS (40 μg/mL) for 6 hours and then collected for qRT-PCR analysis. *P < 0.05. (*n* = 3). (B) Immunoblot analysis for phosphorylated STAT3 (p-STAT3) and total STAT3 (T-STAT3) in total protein pools from WT and miR-149*^−/−^ mouse (KO) livers after LPS (10 mg/Kg body weight) treatment for 16 hours (*n* = 5–6). (**C**) The data of relative protein levels in (B) are expressed as fold change over the ratio of p-STAT3 to T-STAT3 in the control group (lane 1). *P < 0.05, **P < 0.005. (**D**) miR-149* mimics suppressed IL-6-induced p-STAT3 in HepG2 cells. Cells were transfected with miR-149* mimics or control mimics (Scr-miR). Then cells were treated with IL-6 (20 ng/mL) for 6 hours (*n* = 3). (**E**) The data of relative protein levels in (D) are expressed as fold change over the ratio of p-STAT3 to T-STAT3 in the control group (lane 1). *P < 0.05, **P < 0.005. (**F**) miR-149* suppressed STAT3 transactivity induced by LPS. HepG2 cells were cotransfected with miR-149* mimic or control mimic (Scr-miR), the STAT3 reporter plasmid and phRL-TK. After transfection, cells were treated with LPS (40 μg/mL) for 6 hours. *P < 0.05. RLU, relative luciferase units. (G) miR-149* suppressed STAT3 transactivity induced by IL-6. HepG2 cells were cotransfected with miR-149* mimic or control mimic (Scr-miR), the STAT3 reporter plasmid and phRL-TK. After transfection, cells were treated with IL-6 (10 ng/mL) for 6 hours. *P < 0.05 (*n* = 3).

We noticed that miR-149* mimics suppressed STAT3-meidated gene expression from the above results *in vitro* and *in vivo*. Thus, we investigated that whether miR-149* antagonized STAT3-mediated target genes. We found that miR-149* mimics suppressed LPS-induced expression of C3, MMP2 and SOCS3 mediated by STAT3 (Figure 6A), suggesting that miR-149* suppressed inflammation response possibly through antagonizing STAT3 cell signaling. We also used S3I-201, an inhibitor of STAT3 [18], to block STAT3 signaling. It was found that S3I-201 enhanced the inhibition of miR-149* on some proinflammatory gene expression induced by LPS (Supplementary Figure 1)

### Deficiency of miR-149* in liver is more sensitive LPS-induced STAT3 phosphorylation

STAT3 is a major mediator for inflammation [19–21]. In order to reveal the mechanism by which miR-149* regulated inflammatory response in liver, we furthermore investigated the effect of deficiency of miR-149* on STAT3 signaling pathway. We observed that LPS increased 2-fold higher of STAT3 phosphorylation in miR-149*^−/−^ livers than that in WT livers (Figure 6B, 6C). It indicates that miR-149* may be a repressor of STAT3 pathway.

### MiR-149* mimics suppressed the phosphorylation of STAT3 and STAT3 transactivity in HepG2 cells

We used IL-6 to induce STAT3 phosphorylation. IL-6 induced the phosphorylation of STAT3 significantly (Figure 6D, 6E). We found that miR-149* mimics in HepG2 cells inhibited STAT3 phosphorylation induced by IL-6 by about 28% (Figure 6D, 6E).

We next transfected HepG2 cells with a STAT3 reporter plasmid and the control plasmid to assess the effects of the miR-149* mimics on the STAT3 reporter activity. LPS treatment resulted in 1.4-fold higher STAT3 reporter activity (Figure 6F). MiR-149* mimics suppressed STAT3 activity induced by LPS by about 24% (Figure 6F). These results were confirmed using IL-6-induced STAT activity (Figure 6G). IL-6 increased STAT3 transcactivity obviously (Figure 6G) and miR-149* mimics suppressed STAT3 activity induced by IL-6 (Figure 6G). The results indicate that miR-149* can antagonize STAT3 activity at the levels of gene transcription.

## DISCUSSION

miRNAs comprise a large family of small non-coding RNAs that regulate gene expression post-transcriptionally through usually binding to target sites found within the 3’UTR, coding region or even 5’UTR of the targeted mRNAs [8]. miRNAs regulate multiple cell activities such as migration and survival and play a key role in inflammatory diseases and cancer [22, 23]. Many miRNAs activate STAT3 pathway [24, 25], whereas other miRNAs inhibit STAT3-mediated cell signaling [26, 27]. Our current work shows that miR-149* is a potential repressor of STAT3-regualted inflammatory response through antagonizing STAT3 phosphorylation and its transactivity. It is interesting to study the mechanism by which miR-49* suppressed STAT3 signaling. One possibility is that miR-149* may affect IL-6, an upstream regulator of STAT3. It will be worth continuing this study in the future work.

Suppressing the aberrant activation of STAT3 may at least slow down inflammatory diseases and even cancers [28–30]. In the current work, our results shown that miRNA149* has anti-inflammatory properties in the mouse liver at least partially through antagonizing STAT3 cell signaling. Liver inflammation is closely associated with hepatocarcinogenesis. LPS-treated miR-149*^−/−^ mouse liver shows intense liver injury and inflammation compared with WT mice, which indicates that miR-149* may be a potential therapeutic method for treatment of liver cancer.

There are a few reports about the functions of miR-149* in different diseases. Ding et al. reported that miR-149* directly targets and negatively regulates Prdm16 and that inhibition of miR-149-3p stimulates the thermogenic programme of subcutaneous inguinal WAT, leading to increased energy expenditure in mice [16]. Jin et al. reported that miR-149*, as a p53-responsive microRNA, functions as an oncogenic regulator in human melanoma [14]. miRNA-149* may also serve as an oncogenic regulator in T-ALL by negatively regulating JunB [31]. We also tested some gene expression of JUN signaling. It was found that the gene expression of c-Jun and JunB was downregulated in miR-149*^−/−^ mouse liver compared with the WT group (Supplementary Figure 2), which was different from the report of Fan et al. [31]. It suggests that miR-149* regulates JUN signaling in a specific tissue- or cell-type-dependent manner. However, Lin et al. found that miR-149* induces apoptosis by inhibiting Akt1 and E2F1 in human cancer cells [32]. There is no report about miR-149* in liver inflammation response except that El-Guendy et al. demonstrated that miR-149* is upregulated in hepatitis C virus-infected Egyptian patients [33]. Our results reveal the suppression function of miR-149* in inflammation *in vitro* and *in vivo*, suggesting that miR-149* may be a potential protector in liver inflammation. We noted that miR-149* suppressed specific sets of STAT3 target genes, but not all the target genes, with or without LPS treatment. Ye et al., Huan et al., Wang et al. and Jiang et al. reported that the functions of miRNAs are specific to the type of cell, tissue, disease, condition and the stage of disease development [34–37]. In the current work, our results from Figures 3, 4 and 5 show that miR-149* regulated different STAT3 target gene sets in the different conditions, which may be due to the possibility that the suppression of miRNA-149* on STAT3 target gene expression are in a condition-dependent manner.

In conclusion, our work shows that miR-149* is a repressor of STAT3-mediated hepatic inflammation. These findings indicate that miR-149* is a potential therapeutic target for treatment of inflammation, and its mimic or agomir offers possible therapies for preventing and treating inflammatory-associated liver diseases.

## MATERIALS AND METHODS

### Reagents and Plasmids

Lipopolysaccharide (LPS, from Escbricbia coli 0111:B4) was purchased from Sigma Chemical (St Louis, MO, USA). Interleukin-6 (IL-6) was purchased from PeproTech. The pSTAT3-LUC expression vectors were created in our laboratory. The phRL-TK vector was kindly provided by Xufeng Chen and Akio Kruoda (both City of Hope, Duarte, CA, USA), respectively.

### Injection of sgRNA and Cas9 mRNA

Superovulated female B6D2F1 (C57BL/6 X DBA2) mice (7–8 weeks old) were mated to B6D2F1 males, and fertilized embryos were collected from oviducts. Cas9 mRNA (50 ng/ul) and sgRNA (25 ng/ul) was injected into the cytoplasm of fertilized eggs with well recognized pronuclei in M2 medium (Sigma). The injected embryos were cultured in KSOM with amino acids at 37°C under 5% CO2 in air until blastocyst stage by 3.5 days. Thereafter, 15–25 blastocysts were transferred into uterus of pseudopregnant females.

### T7EI assay analysis for genome modification

Genomic DNA from transgenic mouse ears was extracted using the hexadecyltrimethylammonium bromide (CTAB) method (40) and further used for polymerase chain reaction (PCR) amplification with specific primers (F, TCCAAAGCCTCAGTGATAATGTGC; R, AGATTGGCAAGCAGGG AAAGG). The PCR products were used for the T7 Endonuclease I (T7EI) assay. Briefly, the T7EI assay is as follows: PCR products (a mixture from mutant and wild type) were purified with Qiagen PCR purification kit. They were heated at 95°C for 5 min and then cooled from 95°C to room temperature. The denatured and annealed PCR products were digested with three units of T7EI for 30 min at 37°C and subjected to 2% agarose gel electrophoresis.

### TA cloning and sequencing

PCR products from the founder were cloned using TA cloning kit (Takara) following the manufacturer’s instructions to identify the modifications of founders. Four colonies were picked from each transformation.

### Animals

MiR-149*^+/−^ mice were created by Beijing Nuolanxin Biochemical Technology Co Ltd. For other mouse experiments, eight-week-old wild-type (WT) (C57BL/6J) and miR-149*^−/−^ female mice (on C57BL/6J background) were maintained in a pathogen-free animal facility under a standard 12-hour light-dark cycle. Mice were fasted overnight and then injected intraperitoneally (i.p.) with a single dose of LPS (10 mg/Kg body weight) or phosphate-buffered saline (PBS), followed by feeding water ad libitum. Sixteen hours after the injection, mice were killed by CO_2_ asphyxiation, and the liver was removed for further analysis. The animal study proposal was approved by Henan University Institutional Animal Care and Use Committee (IACUC). All animal experiments were carried out in accordance with an approved Henan University IACUC protocol.

### Administration of miR-149* agomir

The mice were given a tail vein injection with miR-149* agomir (40 nmol/20 g body weight) or miRNA negative control (Ribo-bio, Guangzhou, China). After 48 hours of injection, mice were then injected i.p. with a single dose of LPS (20 mg/Kg body weight) or phosphate-buffered saline (PBS). Six hours later, liver were collected for further analysis.

### Cell culture and transfection

Human hepatoblastoma cells (HepG2) were seeded into 6-well plates (1 × 10^6^ cells/well) and grown in complete culture medium [high-glucose Dulbecco’s modified Eagle’s medium (with L-glutamine) supplied with 10% (vol/vol) inactivated fetal calf serum and 1% (vol/vol) antibiotics–antimycotics] as described [38]. The following day, cells were transfected with miR-149* mimics (25nM) or negative control mimics using Lipofectamine 2000 (Invitrogen, Carlsbad, CA, USA). Eighteen hours after treatment, the cells were treated with LPS (40 μg/mL) and then collected for RNA isolation after a 6-hour incubation. For luciferase assay, cells were co-transfected with pSTAT3-LUC, the control thymidine kinase driven Renilla luciferase plasmid (phRL-TK) with/without miR-149* mimics (25 nM) or negative control mimics. Eighteen hours after transfection, cells were treated with IL-6 (10 ng/mL) for 6 hours. Then cells were harvested and the luciferase activity was determined using a dual-luciferase reporter assay system in accordance with the manufacturer’s instructions (Promega, Madison, WI, USA). Luciferase activities were normalized via cotransfection of phRL-TK. Data are expressed as relative fold activation to that of nonstimulated (−) sets.

### RNA isolation and quantitative real-time polymerase chain reaction

Total RNA was extracted from mouse liver or HepG2 cells using Tri-Reagent (Molecular Research Center, Inc., Cincinnati, OH). Quantitative real-time PCR was performed using the Power SYBR Green PCR Master Mix protocol (Applied Biosystems, Foster City, CA). Amplification of β-actin (for cells) or 36B4 (for mouse liver) was used as an internal reference. Quantitative PCR analysis was conducted using the ABI 7300 Sequence Detection System. Primers sequences are available on request.

### Immunoblot analysis

Cells were transfected with miR-149* mimics (25 nM) or negative control mimics. Then cells were treated with IL-6 (20 ng/mL) for 6 hours. The cells were lysed for immunoblot analysis as reported previously [39–41]. Bands on blots were visualized using Tanon 5200 enhanced chemiluminescence (ECL) detection system (Tanon, China) and quantified with a computerized digital imaging system using Tanon software.

### Analysis of aspartate aminotransferase activity and liver histology

Aspartate aminotransferase activity analysis, terminal deoxynucleotidyl transferase-mediated dUTP nick-end labeling (TUNEL) staining were performed as described previously [41]. Ki67 immunohistochemical staining was performed using a mouse monoclonal anti-mouse Ki67 antibody (Abcam) as described previously [42].

### Statistical analysis

All data represent at least three independent experiments and are expressed as the mean ± standard deviation. The Student’s *t*-test was used to calculate *P* values, unless stated otherwise. For multiple comparisons between groups, a one-way analysis of variance (ANOVA) was performed. A *P* value less than 0.05 was considered significant.

## SUPPLEMENTARY MATERIALS FIGURES


