# Supplementary material for: MicroRNA-149* suppresses hepatic inflammatory response through antagonizing STAT3 signaling pathway
Source: Oncotarget. 2017 Jun 16;8(39):65397–406. doi: 10.18632/oncotarget.18541 (PMC5630339; doi:10.18632/oncotarget.18541)
Supplement: Supplementary file 1 [file oncotarget-08-65397-s001.pdf]

MicroRNA-149* suppresses hepatic inflammatory response through antagonizing STAT3 signaling pathway

SUPPLEMENTARY MATERIALS

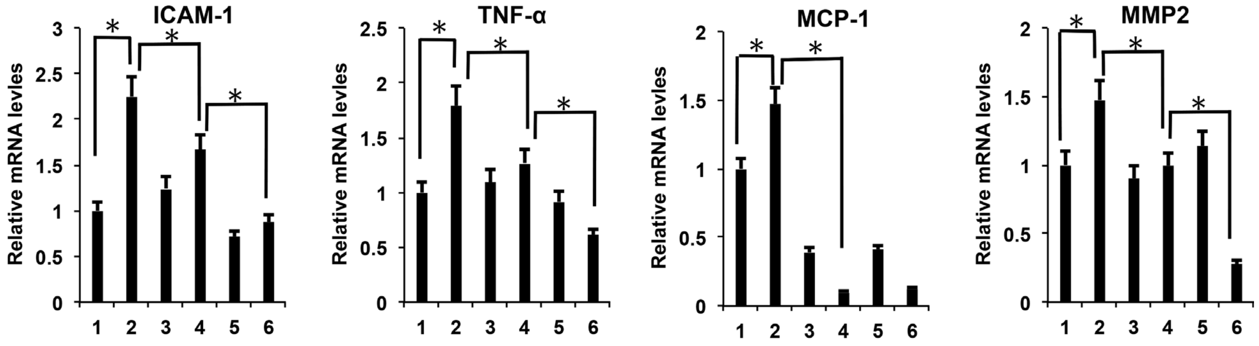

1. Scr-miR
2. Scr-miR+LPS
3. miR-149* mimic
4. miR-149* mimic +LPS
5. miR-149* mimic+S3I-201
6. miR-149*+LPS+S3I-201

Supplementary Figure 1: STAT3 inhibitor S3I-201 enhanced the suppression effect of miR-149* on proinflammatory gene expression induced by LPS. HepG2 cells were transfected with miR-149* mimics or control mimics (Scr-miR). After 24 hours, cells were treated with S3I-201 (100 μM) for 24 hours. Then cells were treated with LPS (40 μg/mL) for 6 hours and collected for qRT-PCR analysis. *$P < 0.05$. ($n = 3$).

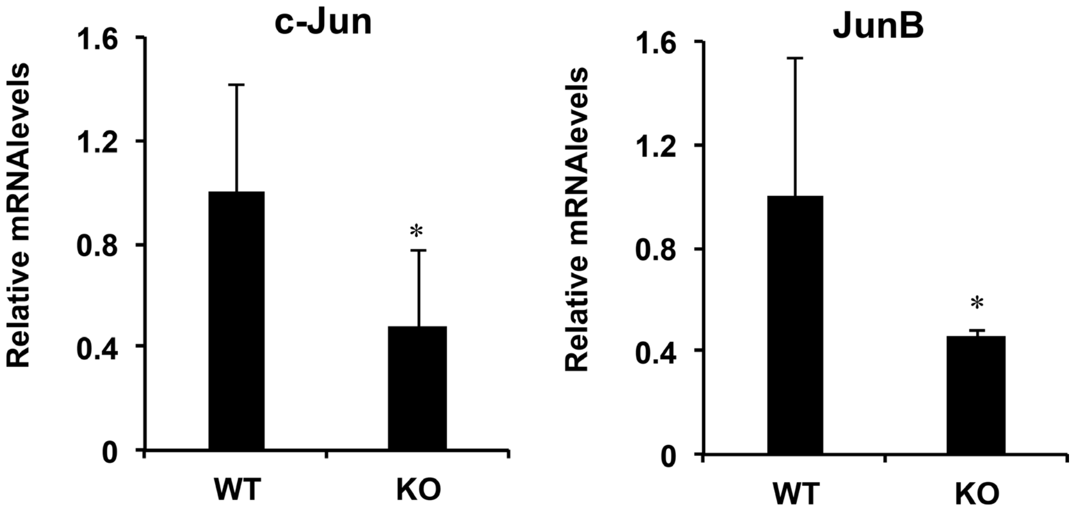

Supplementary Figure 2: The gene expression of c-Jun and JunB in Jun signaling. Quantitative real-time PCR analysis of the expression of c-Jun and JunB in livers from 8-week-old wild-type (WT) or miR-149$^{*-/-}$ (KO) mice (n = 5). *$P < 0.05$ versus the WT group.
